# Characterization of Perturbing Actions by Verteporfin, a Benzoporphyrin Photosensitizer, on Membrane Ionic Currents

**DOI:** 10.3389/fchem.2019.00566

**Published:** 2019-08-22

**Authors:** Mei-Han Huang, Ping-Yen Liu, Sheng-Nan Wu

**Affiliations:** ^1^College of Medical and Health Sciences, Fooyin University, Kaohsiung City, Taiwan; ^2^Division of Cardiovascular Medicine, National Cheng Kung University Medical College, Tainan City, Taiwan; ^3^Institute of Basic Medical Sciences, National Cheng Kung University Medical College, Tainan City, Taiwan; ^4^Department of Physiology, National Cheng Kung University Medical College, Tainan City, Taiwan

**Keywords:** verteporfin, Ca^2+^-activated K^+^ current, K^+^ current, BKCa channel, Ca^2+^ current, pituitary tumor cell, glioma cell

## Abstract

Verteporfin (VP), a benzoporphyrin derivative, has been clinically tailored as a photosensitizer and recently known to suppress YAP-TEAD complex accompanied by suppression of the growth in an array of neoplastic cells. However, the detailed information is little available regarding possible modifications of it and its related compounds on transmembrane ionic currents, despite its growing use in clinical settings. In this study, from whole cell recordings, VP (0.3–100 μM) increased the amplitude of Ca^2+^-activated K^+^ currents (*I*_K(Ca)_) in pituitary tumor (GH_3_) cells in a concentration-dependent manner with an EC_50_ value of 2.4 μM. VP-stimulated *I*_K(Ca)_ in these cells was suppressed by further addition of either paxilline, iberiotoxin, or dithiothreitol, but not by that of tobultamide or TRAM-39. VP at a concentration of 10 μM mildly suppressed the amplitude of delayed-rectifier K^+^ current; however, it had minimal effects on M-type K^+^ current. In cell-attached current recordings, addition of VP to the recording medium enhanced the activity of large-conductance Ca^2+^-activated K^+^ (BK_Ca_) channels. In the presence of VP, additional illumination with light intensity of 5.5 mW/cm^2^ raised the probability of BK_Ca_-channel openings further. Addition of VP decreased the peak amplitude of L-type Ca^2+^ current together with slowed inactivation time course of the current; however, it failed to modify voltage-gated Na^+^ current. Illumination of GH_3_ cells in continued presence of VP also induced a non-selective cation current. Additionally, VP increased the activity of BK_Ca_ channels in human 13-06-MG glioma cells with an EC_50_ value of 1.9 μM. Therefore, the effects of VP on ionic currents described herein tend to be upstream of its inhibition of YAP-TEAD complex and they are conceivably likely to contribute to the underlying mechanisms through which it and its structurally similar compounds effect the modifications in functional activities of pituitary or glial neoplastic cells, if the *in vivo* findings occur.

## Introduction

Verteporfin (VP, Visudyne®), a benzoporphyrin derivative, is a compound therapeutically tailored as a photosensitizer for photodynamic therapy, because this agent can effectively eliminate aberrant blood vessels in the eye associated with conditions such as either the wet form of macular degeneration or abnormal choroidal neovascularizations (Schmidt-Erfurth and Hasan, 2000; DeRosa and Crutchley, 2002; Renno et al., 2004; Rosenblatt et al., 2005; Hua et al., 2014; Hu et al., 2015; Chen and Hu, 2017; Ghazai et al., 2017; Konstantinou et al., 2017; AlAmri et al., 2018; Baskaran et al., 2018; Liu et al., 2018; Min et al., 2018; Iacono et al., 2019; Isildak et al., 2019).

Yes-associated protein (YAP) has been reported to be a main mediator of the Hippo pathway, which is thought to promote cancer development (Feng et al., 2016; Gibault et al., 2016; Kandoussi et al., 2017; Abe et al., 2018; Chen et al., 2019). A number of recent reports has demonstrated that VP can suppress the aberrant growth in a variety of tumor cell lines, including breast cancer, glioma, ovarian cancer, hepatocellular carcinoma, and retinoblastoma, through an intriguing mechanism linked to specific suppression of YAP-TEAD complex (Valero et al., 2015; Feng et al., 2016; Al-Moujahed et al., 2017; Gibault et al., 2017; Abe et al., 2018; Mulder et al., 2018; Li et al., 2019; Pellosi et al., 2019; Qin et al., 2019; Zhang et al., 2019). This compound has been indeed long supposed to be an inhibitor of YAP-TEAD complex and hence to play the roles in growth inhibition in different types of neoplastic cells including pituitary tumors and gliomas (Feng et al., 2016; Gibault et al., 2016, 2017; Al-Moujahed et al., 2017; Deng et al., 2018; Eales et al., 2018; Liao et al., 2018; Pellosi et al., 2019; Zhang et al., 2019).

The photodynamic therapy with different photosensitizers such as hypericin or VP has been previously demonstrated to be effective in the treatment of different types of hyper- or neoplastic tissues, including the residual small tumor in pituitary gland (Faustino et al., 1997; Marks et al., 2000; Valenzeno and Tarr, 2001; DeRosa and Crutchley, 2002; Rahimipour et al., 2003; Brown et al., 2004; Renno et al., 2004; Solban et al., 2006; Triesscheijn et al., 2006; Zhou et al., 2006; Cole et al., 2008; Tekrony et al., 2011; Nemes et al., 2016; Deng et al., 2018). Previous work indeed reported the capability of photosensitizers (e.g., rose bengal) to modify membrane ionic current in pituitary tumor (GH_3_) cells or heart cells (Tarr et al., 1994; Valenzeno and Tarr, 1998, 2001). Alternatively, earlier reports have also revealed that VP could induce anterior ischemic optic neuropathy in rodents (Karacorlu et al., 2004; Min et al., 2018). However, surprisingly, little information has been thus far available concerning any possible modifications of VP on the level of surface membrane including ionic channels in a variety of neoplastic cells, despite its clinical approval for photodynamic therapy.

Because of the considerations described above, we sought to determine whether VP and its related compounds could exert any possible perturbations on different types of ionic currents which include Ca^2+^-activated K^+^ current (*I*_K(Ca)_), delayed-rectifier K^+^ current (*I*_K(DR)_), M-type K^+^ current (*I*_K(M)_), large-conductance Ca^2+^-activated K^+^ (BK_Ca_) channel, L-type Ca^2+^ current (*I*_Ca,L_), and voltage-gated Na^+^ current (*I*_Na_). In this study, we provide substantial evidence to unravel that VP is indeed capable of modifying membrane ionic currents in pituitary tumor (GH_3_) cells and in glioma (13-06-MG) cells, the actions of which are apparently of clinical relevance and appear to be upstream of its inhibition at YAC-TEAD complex.

## Materials and Methods

### Drugs and Solutions

Verteporfin (VP, Visudyne®, BPD-MA, CL-318,952, C_41_H_42_N_4_O_8_,), rose bengal, tetraethylammonium chloride (TEA), tetrodotoxin (TTX), and tolbutamide were acquired from Sigma-Aldrich (St. Louis, MO), iberiotoxin and paxilline from Alomone (Jerusalem, Israel), and A-803467 (5-(4-chlorophenyl)-*N*-(3,5-dimethoxyphenyl)-2-furancarboxamide), A-887826 (5-(4-butoxy-3-chlorophenyl)-*N*-[[2-(4-morpholinyl)-3-pyridinyl]methyl-3-pyridine carboxamide), 2-guanidine-4-methylquinazoline (GMQ), S(-)-Bay K 8644 (Bay K 8644), nifedipine, NS1619, PF573228 (3,4-dihydro-6-[[4-[[[3-(methylsulfonyl)phenyl]methyl]amino]-5-(trifluoromethyl)-2-pyrimidinyl]amino]-2(1H)-quinolinone) and TRAM-39 (2-cholo-α,α-diphenylbenzeneacetonitrile) were from Tocris Cookson Ltd. (Bristol, UK), and pioglitazone was obtained from Takeda (Tokyo, Japan). Chlorotoxin was kindly provided by Professor Dr. Woei-Jer Chuang, Department of Biochemistry, National Cheng Kung University Medical College, Tainan, Taiwan. To protect VP from light, stock solution containing this compound was wrapped in aluminum foil. Cell culture media were obtained from Invitrogen (Carlsbad, CA), unless stated otherwise, and other chemicals or solvents such as CdCl_2_, CsCl, CsOH, and *N*-methyl-D-glucamine^+^ (NMDG^+^) were of analytical reagent grade. The twice-distilled water that had been de-ionized through a Millipore-Q system was used in all experiments.

The composition of bath solution (i.e., HEPES-buffered normal Tyrode's solution) for GH_3_ or 13-06-MG cells was as follows (in mM): NaCl 136.5, KCl 5.4, CaCl_2_ 1.8, MgCl_2_ 0.53, glucose 5.5, and HEPES-NaOH buffer 5.5 (pH 7.4). To measure K^+^ currents [i.e., *I*_K(Ca)_*, I*_K(DR)_, and *I*_K(M)_], we filled the patch pipette with a solution (in mM): KCl 140, MgCl_2_ 1, Na_2_ATP 3, Na_2_GTP 0.1, EGTA 0.1, and HEPES-KOH buffer 5 (pH 7.2); however, in some separate set of experiments of reducing the level of intracellular Ca^2+^, EGTA concentration in the solution was changed to 10 mM. To record *I*_K(M)_, high K^+^-bathing solution was composed of the following (in mM): KCl 145, MgCl_2_ 0.53, and HEPES-KOH 5 (pH 7.4). In order to measure *I*_Na_, *I*_Ca,L_, or *I*_NS_ precisely, we replaced K^+^ ions inside the pipette solution with equimolar Cs^+^ ions, and pH was then appropriately adjusted to 7.2 with CsOH. In order to fully suppress Na^+^, Ca^2+^ or K^+^ currents, TTX (1 μM), CdCl_2_ (0.5 mM) or TEA (10 mM) was added to the recording medium, respectively. The pipette solution and culture medium were commonly filtered on the day of use with Acrodisc® syringe filter with 0.2 μm Supor® membrane (Pall Corp., Port Washington, NY).

### Cell Culture

GH_3_ pituitary tumor cells, obtained from the Bioresources Collection and Research Center ([BCRC-60015]; Hsinchu, Taiwan), were maintained in Ham's F-12 media supplemented with 15% horse serum (v/v), 2.5% fetal calf serum (v/v), and 2 mM L-glutamine (Wu et al., 2003; Lin et al., 2014; So et al., 2018). The glioblastoma multiforme cell line (13-06-MG) was kindly provided by Professor Dr. Carol A. Kruse, Department of Neurosurgery, Ronald Reagan UCLA Medical Center, LA, CA, USA. Cells were grown in high-glucose (4 g/l) Dulbecco's modified Eagle media supplemented with 10% heat-inactivated fetal bovine serum (Huang et al., 2015). GH_3_ or 13-06-MG cells were maintained at 37°C in a humidified environment of 5% CO_2_/95% air and sub-cultured weekly and fresh media were generally added every 2–3 days to maintain a healthy cell population. Glial cells were verified by identifying glial fibrillary acidic protein, a cytoskeletal protein. The experiments were performed after 5 or 6 days of subcultivation (60–80% confluence).

### Electrophysiological Measurements

Shortly before experimentation, we dissociated the cells and then placed a few drops of cell suspension onto a custom-built recording chamber affixed on the stage of a DM-IL inverted microscope (Leica, Wetzlar, Germany). As being settled down the bottom of the chamber, cells were bathed at a room temperature of 20–25°C in normal Tyrode's solution, the composition of which is describe above. Once cells were placed in the VP-containing medium, the recordings were carried out under very dim room light. For illumination system, we focused visible light onto a spot on the recording chamber which covered completely the area of cells exposed to VP in the central region with an illumination intensity of ~5.5 mW/cm^2^. We fabricated the recording pipette from Kimax-51 capillary tubes (#34500; Kimble, Vineland, NJ) using a vertical PP-83 (Narishige, Tokyo, Japan) or a horizontal P-97 Flaming/Brown (Sutter, Novato, CA) puller, and their tips were fire-polished with an MF-83 microforge (Narishige). During the measurements, the pipette with a resistance of 3–5 MΩ, which was inserted into holder, was gently maneuvered by using a WR-98 micromanipulator (Narishige). Patch-clamp experiments were measured in either cell-attached, inside-out, or whole-cell arrangement by using an RK-400 patch-clamp amplifier (Bio-Logic, Claix, France) connected with a personal computer with 64-bit processor (Lin et al., 2004; Wu et al., 2017; So et al., 2019). Shortly before giga-seal formation was achieved, the potentials were appropriately corrected for the liquid junction potential that commonly develop at the pipette tip as the composition of the pipette solution was quite different from that in the bath. Tested compounds were applied by perfusion or added to the bath to obtain the final concentration indicated.

### Data Recordings

The data comprising both potential and current traces were stored online in an ASUS VivoBook Flip-14 touchscreen laptop computer (TP412U; Taipei City, Taiwan) at 10 kHz equipped with the 12-bit Digidata 1440A interface (Molecular Devices, Inc., Sunnyvale, CA). The latter device was used for efficient analog-to-digital/digital-to-analog conversion. During the experiments, data acquisition system was electronically driven by pCLAMP 10.7 software (Molecular Devices) run under Windows 10 (Redmond, WA), and the signals were simultaneously monitored on LCD monitor (MB169B+; ASUS, Taipei, Taiwan) through a USB type-C connection. Current signals were low-pass filtered at 2 kHz with FL-4 four-pole Bessel filter (Dagan, Minneapolis, MN) to minimize background noise. Through digital-to-analog conversion, the pCLAMP-generated voltage-clamp profiles with different waveforms were applied to determine the current-voltage (*I-V*) relationships for different types of ionic currents such as *I*_K(Ca)_, *I*_K(DR)_, or *I*_Ca,L_. When high-frequency stimuli used to elicit the cells were required, an Astro-med Grass S88X dual output pulse stimulator (Grass Technologies, West Warwick, RI) was used.

### Data Analyses

To evaluate concentration-dependent effect of VP on the stimulation of *I*_K(Ca)_ in GH_3_ cells, each cell was depolarized to +50 mV from a holding potential of 0 mV. Current amplitude measured at the end of each depolarizing pulse during cell exposure to 100 μM VP was taken to be 100%, and those in the presence of different VP concentrations were then compared. To determine concentration-dependent stimulation of VP on BK_Ca_ channels recorded from human 13-06-MG glioma cells, channel activity at +60 mV relative to the bath during the exposure to 100 μM VP was considered as 100%, and the channel open probabilities at different VP concentrations were compared. The mean values for concentration-dependent relation of VP on the stimulation of *I*_K(Ca)_ or BK_Ca_ channels were least-squares fitted to a modified Hill function. That is,

$$\begin{array}{l} Percentage\;increase\;\left(\%\right)=\frac{E_{\max}}{1+\;\frac{EC_{50}^{n_{H}}}{{\left[VP\right]}^{n_{H}}}}, \end{array}$$

where [VP] is the VP concentration applied, EC_50_ the concentration required for half-maximal stimulation of *I*_K(Ca)_ or BK_Ca_ channels, n_H_ the Hill coefficient, and *E*_max_ VP-induced maximal stimulation of either *I*_K(Ca)_ in GH_3_ cells or BK_Ca_ channels in 13-06-MG cells.

Single-BK_Ca_ channel currents were analyzed by pClamp 10.7 software (Molecular Devices). Multi-gaussian adjustments of the amplitude distributions among channels were commonly used to determine single-channel currents. Functional independence between channels was verified by comparing the observed stationary probabilities with the values calculated according to the binomial law. The open-state probability of the channels was expressed as *N*·*P*_O_ which can be estimated using the following equation:

$$\begin{array}{l} N\cdot P_{O}=\frac{A_{1}+2A_{2}+3A_{3}+\ldots +nA_{n}}{A_{0}+A_{1}+A_{2}+A_{3}+\ldots +A_{n}}, \end{array}$$

where *N* is the number of active channels in the patch examined, *A*_0_ the area under the curve of an all-points histogram corresponding to the closed state, and *A*_1_-*A*_n_ are the histogram areas reflecting the levels of distinct open state for 1 to n channels in the patch.

### Statistical Analyses

To perform linear or non-linear (e.g., sigmoidal or exponential function) curve fitting to the data set (i.e., the goodness of fit) was appropriately utilized by using OriginPro (OriginLab, Northampton, MA) or Prism version 5.0 (GraphPad, La Jolla, CA). Data were analyzed and are plotted using OriginPro (OriginLab), and they were expressed as mean±standard error of the mean (SEM). The paired or unpaired Student's *t*-test, or one-way analysis of variance (ANOVA) followed by *post-hoc* Fisher's least-significance difference test for multiple comparisons, were implemented for the statistical evaluation of differences among means. We further used non-parametric Kruskal-Wallis test, as the assumption of normality underlying ANOVA was likely to be violated. Statistical analyses were commonly performed by using IBM SPSS® version 20.0 (IBM Corp., Armonk, NY). *P* < 0.05 was considered significant, unless stated otherwise.

## Results

### Stimulatory Effect of VP on Ca^2+^-Activated K^+^ (I_**K(Ca)**_) Measured From Pituitary Tumor (GH_3_) Cells

**Figure 1:**
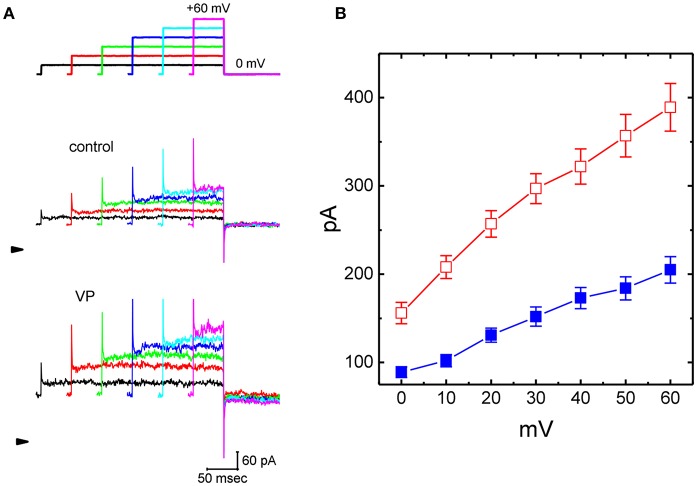
Effects of verteporfin (VP) on Ca^2+^-activated K^+^ current (*I*_K(Ca)_) measured from pituitary GH_3_ cells. In these experiments, cells were bathed in normal Tyrode's solution containing 1.8 mM CaCl_2_, and the recording pipette was filled with K^+^-containing solution. The examined cell was voltage clamped at 0 mV to inactivate most of other types of voltage-gated K^+^ currents, and a series of the voltage steps ranging between 0 and +60 mV [indicated in the uppermost part of **(A)**] was delivered. **(A)** Representative *I*_K(Ca)_ traces taken in the absence (upper) and presence (lower) of 3 μM VP. The arrowhead indicates the zero current level, and calibration bar in the bottom right corner refers to both panels. **(B)** Averaged current-voltage (*I-V*) relationships of *I*_K(Ca)_ measured from the control (■) and during cell exposure to 3 μM VP (□) (mean ± SEM; *n* = 11 for each point).

The whole cell configuration of the patch-clamp technique was initially carried out to evaluate any possible perturbations of VP on *I*_K(Ca)_ amplitude in these cells. In the experiments designed to measure *I*_K(Ca)_, we immersed the cells in normal Tyrode's solution containing 1.8 mM CaCl_2_, and the recording pipette was filled with a solution which contained a low concentration 140 mM K^+^, 0.1 mM EGTA, and 3 mM ATP. As whole-cell mode was achieved, the examined cell was maintained at the level of 0 mV to inactivate other types of voltage-gated K^+^ currents which are enriched in these cells (Stojilkovic et al., 2010; So et al., 2018), and ionic currents in response to a series of the voltage pulses between 0 and +60 mV were robustly elicited. Current amplitude was progressively increased as the test voltage became more positive (Figure 1). When extracellular Ca^2+^ was removed, current amplitudes were greatly reduced; meanwhile, an increase in intracellular EGTA concentration from 0.1 to 10 mM also abolished these currents. This type of outward currents with an outwardly rectifying property has been hence regarded as *I*_K(Ca)_ (Lin et al., 2004; Wu et al., 2017), the amplitudes of which become greatly small as intracellular Ca^2+^ level is decreased (i.e., intracellular EGTA with 10 mM).

As cells were exposed to VP, the amplitude of *I*_K(Ca)_ in response to depolarizing step was considerably increased (Figures 1A,B). For example, VP at a concentration of 3 μM significantly raised *I*_K(Ca)_ measured at the level of +50 mV from 184 ± 13 to 257 ± 24 pA (*n* = 11, *P* < 0.05). Washout of VP, current amplitude returned to 197 ± 14 pA (*n* = 10, *P* < 0.05). Similarly, as cells were exposed to 10 μM NS1619, an activator of BK_Ca_ channels, *I*_K(Ca)_ amplitude at +50 mV increased from 179 ± 14 to 235 ± 25 pA (*n* = 8, *P* < 0.05). Figure 1B illustrates the averaged current-voltage (*I-V*) relationships of *I*_K(Ca)_ with or without addition of VP (3 μM).

**Figure 2:**
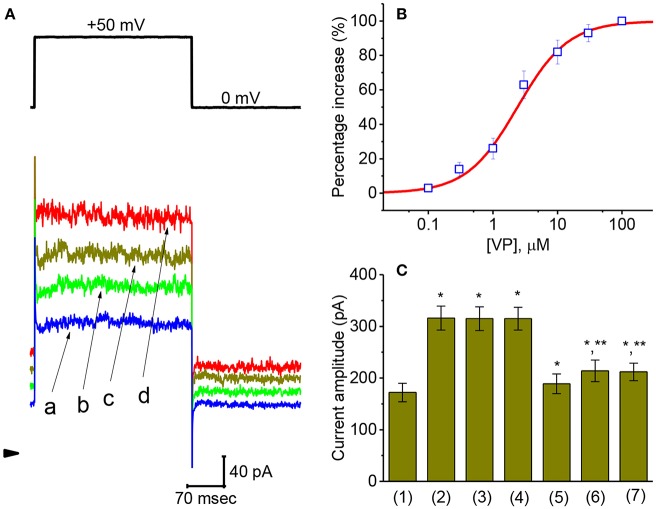
Concentration-dependent effect of VP on *I*_K(Ca)_ and the effects of different related compounds on VP-stimulated *I*_K(Ca)_ in GH_3_ cells. The experiments were conducted in cells bathed in normal Tyrode's solution and the depolarizing pulse from 0 to +50 mV [indicated in the upper part of **(A)**] was delivered to the cell examined. **(A)** Superimposed *I*_K(Ca)_ taken during step depolarization. a: control; b:1 μM VP; c: 3 μM VP; d: 10 μM VP. Arrowhead is the zero-current level and calibration bar refers to all traces. **(B)** Concentration-dependent effect of VP on *I*_K(Ca)_ amplitude [mean ± SEM; *n* = 10–14 for each point (□)]. The examined cell was depolarized from 0 to +50 mV and current amplitude at the end of each depolarizing step was measured. As cells were exposed to 100 μM VP, *I*_K(Ca)_ amplitude was taken to be 100%, and those at different VP concentrations were then compared. The values for EC_50_ and the Hill coefficient were 2.4 μM and 1.1, respectively. Non-linear smooth line was least-squares fitted to a modified Hill function described in **Materials and Methods**. **(C)** Summary bar graph showing the effect of VP, VP plus tolbutamide, VP plus TRAM-39, VP plus paxilline, VP plus iberiotoxin, and VP plus dithiothreitol. Each bar showing *I*_K(Ca)_ amplitude at the level of +60 mV indicates the mean ± SEM (*n* = 11–14). *Significantly different from control (*P* < 0.05) and **significantly different from VP (3 μM) alone group (*P* < 0.05).

Concentration-dependent stimulation of *I*_K(Ca)_ on GH_3_ cells by VP was further derived and then constructed. In these experiments, we bathed the cells in normal Tyrode's solution, the examined cell was clamped at 0 mV, and depolarizing pulse from 0 to +50 mV was then delivered. As illustrated in Figures 2A,B, the presence of different VP concentrations (0.1–100 μM) could be efficacious at raising *I*_K(Ca)_ amplitude in a concentration-dependent manner. According to least-squares minimization procedure, the half-maximal concentration required for the stimulatory effect of VP on *I*_K(Ca)_ amplitude was estimated to be 2.4 μM, and it at a concentration of 100 μM fully increased current amplitude elicited by voltage depolarization. The experimental results thus demonstrate that VP has a stimulatory action on *I*_K(Ca)_ seen in GH_3_ cells in a concentration-dependent manner.

### Comparison of Effects of VP, VP Plus Tolbutamide, VP Plus TRAM-39, VP Plus Paxilline, VP Plus Iberiotoxin, and VP Plus Dithiothreitol on I_**K(Ca)**_ Amplitude in GH_3_ Cells

An earlier work has demonstrated that VP might disrupt mitochondrial inner membrane potential in cancer cells (Belzacq et al., 2001). On the other hand, the activity of different types of K^+^ channels present in pituitary cells including GH_3_ cells (Stojilkovic et al., 2010) might contribute synergistically to VP-mediated effect on *I*_K(Ca)_. Therefore, during cell exposure to VP, subsequent addition of different compounds including tolbutamide, TRAM-39, paxilline, iberiotoxin, and dithiothreitol was further studied to evaluate whether those agents exert any modulation on *I*_K(Ca)_ stimulated by VP. As depicted in Figure 2C, subsequent application of neither tolbutamide nor TRAM-39 could attenuate VP-mediated increase of *I*_K(Ca)_ amplitude; however, that of paxilline, iberiotoxin, or dithiothreitol did reduce *I*_K(Ca)_ raised by VP. Tolbutamide and TRAM-39 are blockers of ATP-sensitive K^+^ (K_ATP_) and intermediated-conductance Ca^2+^-activated K^+^ (IK_Ca_) channels (Chen P. C. et al., 2018; Chen T. S. et al., 2018; Liu et al., 2019a), respectively, while paxilline and iberiotoxin could suppress the activity of large-conductance Ca^2+^-activated K^+^ (BK_Ca_) channels, respectively. Dithiothreitol is recognized as a sulfhydryl reducing agent.

### Inhibitory Effect of VP on Delayed-Rectifier K^+^ Current (I_**K(DR)**_) in GH_3_Cells

**Figure 3:**
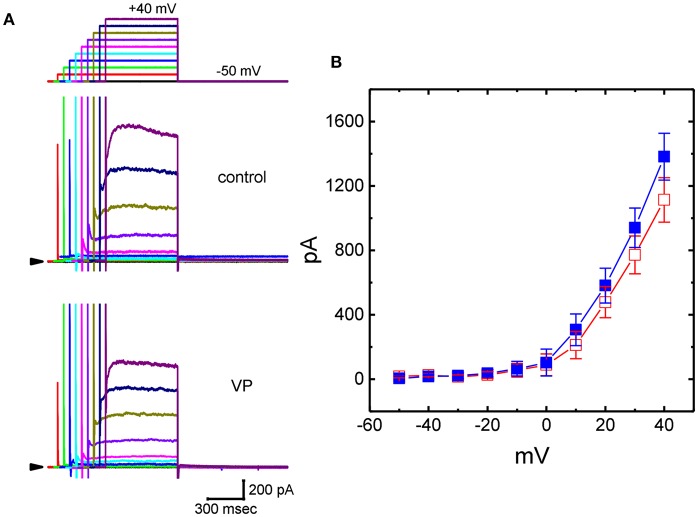
Effects of VP on delayed-rectifier K^+^ current (*I*_K(DR)_) in GH_3_ cells. In these experiments, we bathed cells in Ca^2+^-free Tyrode's solution containing 1 μM tetrodotoxin (TTX), and whole-cell currents were recorded by filling the pipette with K^+^-containing solution. The examined cell was held at −50 mV and a series of depolarizing steps ranging between −50 and +40 mV was applied [indicated in the uppermost part of **(A)**]. **(A)** Superimposed *I*_K(DR)_ traces obtained during the control (upper) and cell exposure to 10 μM VP (lower). Arrowhead in each panel represents the zero-current level. **(B)** Averaged *I-V* relationships for *I*_K(DR)_ obtained with or without the addition of 10 μM VP (mean ± SEM; *n* = 13 for each point). Current amplitudes were measured at the end of each depolarizing step. ■: control; □: in the presence of 10 μM VP.

We further studied whether, in addition to *I*_K(Ca)_, another different types of K^+^ currents [e.g., *I*_K(DR)_] could be modified by the presence of VP. In these experiments, cells were bathed in Ca^2+^-free Tyrode's solution containing 1 μM TTX and 0.5 mM CdCl_2_ and we filled the recording pipette by using K^+^-containing solution. Addition of 3 μM VP was not found to modify *I*_K(DR)_ amplitude significantly. However, as GH_3_ cells were exposed to 10 μM VP, the *I*_K(DR)_ amplitudes elicited in response to stepwise depolarizing voltages were progressively decreased (Figures 3A,B). Figure 3B illustrates averaged *I-V* relationships of *I*_K(DR)_ measured at the end of each depolarizing pulse, as the results were obtained in the absence and presence of 10 μM VP. For example, as the examined cells were depolarized from −50 to +40 mV, addition of 10 μM VP diminished *I*_K(DR)_ amplitude from 1,382 ± 145 to 1,114 ± 138 pA (*n* = 13, *P* < 0.05). After washout of the drug, current amplitude returned to 1,328 ± 141 pA (*n* = 11, *P* < 0.05). However, no modification of *I*_K(DR)_ inactivation time course in response to membrane depolarization was demonstrated in the presence of 10 μM VP. Therefore, these results showed that VP at a concentration of 10 μM mildly suppressed the amplitude of *I*_K(DR)_ in GH_3_ cells.

### Failure of VP to Modify M-Type K^+^ Current (I_**K(M)**_) Recorded From GH_3_Cells

**Figure 4:**
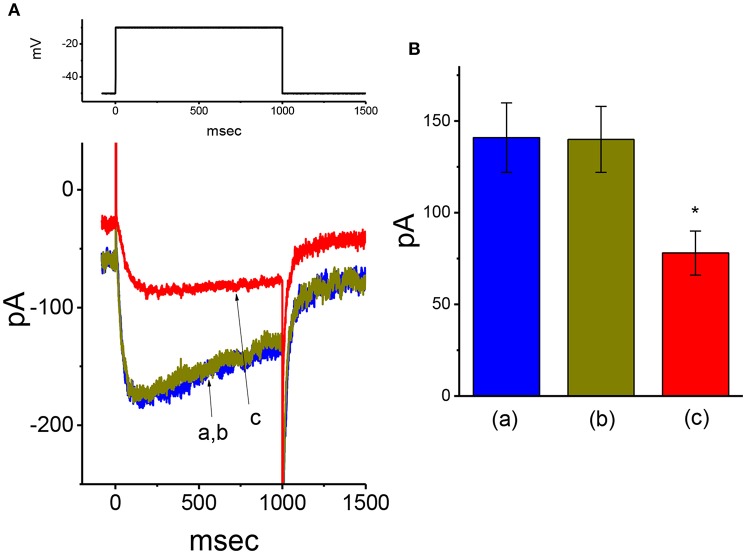
Failure of VP to alter M-type K^+^ current (*I*_K(M)_) recorded from GH_3_ cells. In these experiments, we bathed cells in high-K^+^, Ca^2+^-free solution, and filled the pipette by using K^+^-containing solution. **(A)** Original *I*_K(M)_ traces in response to long-lasting membrane depolarization from −50 to −10 mV. a: control; b: 10 μM VP; c: 10 μM VP plus 3 μM pioglitazone. The voltage protocol used is indicated in the upper part. **(B)** Summary bar graph showing the effect of VP and VP plus pioglitazone on *I*_K(M)_ elicited by membrane depolarization (mean ± SEM; *n* = 11 for each bar). *Significantly different from control (*P* < 0.05).

In addition to *I*_K(DR)_, another type of K^+^ current [i.e., *I*_K(M)_] detected in GH_3_ cells (Sankaranarayanan and Simasko, 1996; Stojilkovic et al., 2010; So et al., 2019) was further designed to evaluate whether the presence of VP has any modifications on this type of K^+^ current. To amplify *I*_K(M)_, we bathed cells in high-K^+^ (145 mM), Ca^2+^-free solution, the composition of which is described in **Materials and Methods**. In these experiments, the examined cell held in voltage clamp at −50 mV with a long-duration depolarizing step to −10 mV was able to generate a slowly activating K^+^ inward current followed by a largely deactivating current, namely *I*_K(M)_, as described previously (Sankaranarayanan and Simasko, 1996; Chen T. S. et al., 2018; Liu et al., 2019b; So et al., 2019). As illustrated in Figure 4, under our experimental conditions, we were unable to find out that the presence of 10 μM VP had any measurable effect on *I*_K(M)_ amplitude [141 ± 19 pA [in the control] vs. 140 ± 18 pA [in the absence of 10 μM VP], *n* = 11, *P* > 0.05]. However, in continued presence of 10 μM VP, further application of 3 μM pioglitazone was effective at suppressing *I*_K(M)_, as evidenced by the results showing that, in continued presence of 10 μM VP, further application of 10 μM pioglitazone substantially decreased *I*_K(M)_ amplitude to 78 ± 12 pA (*n* = 11, *P* < 0.05). Therefore, the data prompted us to indicate that distinguishable from *I*_K(Ca)_ or *I*_K(DR)_, the *I*_K(M)_ observed in GH_3_ cells was relatively resistant to modification by VP, though it was potently suppressed by pioglitazone (Chen T. S. et al., 2018).

### Effects of VP on the Activity of Bk_**ca**_ Channels in GH_3_Cells

**Figure 5:**
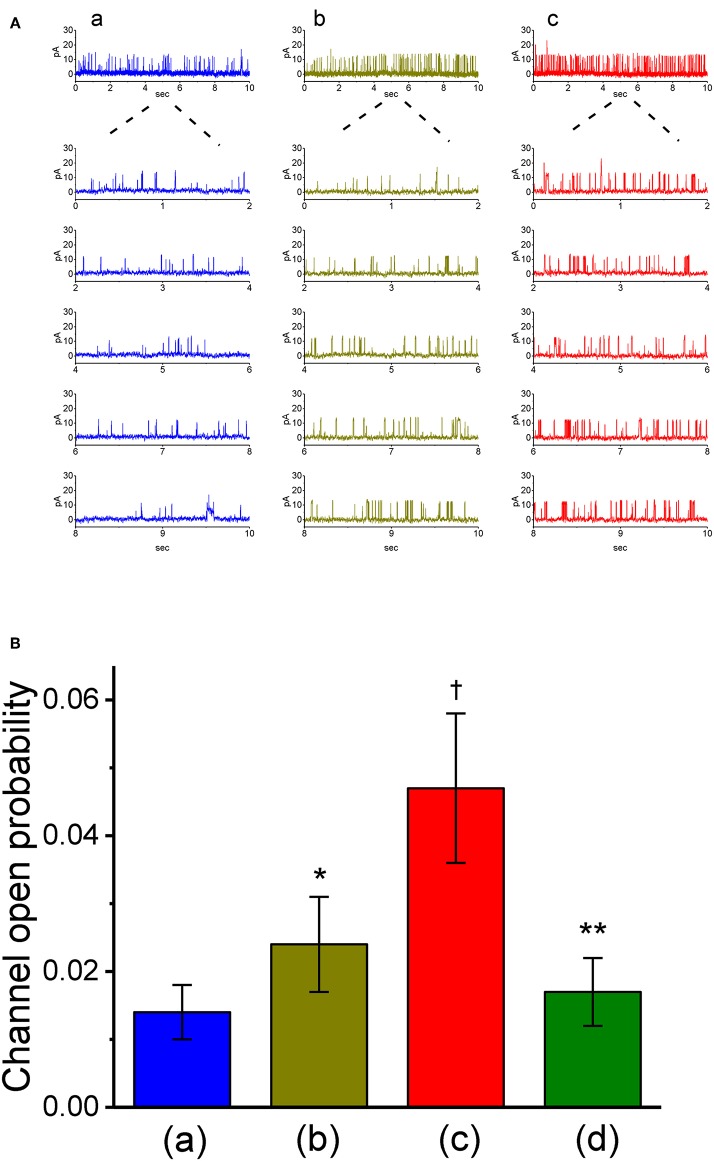
Stimulatory effect of VP on the activity of large-conductance Ca^2+^-activated K^+^ (BK_Ca_) channels measured from on-cell patch recordings of GH_3_ cells. In these cell-attached current recordings, cells were bathed in normal Tyrode's solution containing 1.8 mM CaCl_2_ and channel activity was measured by filling the pipette with K^+^-containing solution. **(A)** BK_Ca_ channel activity measured at the holding potential of +60 mV relative to the bath. Panel a is control (i.e., in the absence of light and photosensitizer VP), while panels b and c were obtained in the presence of 3 μM VP and 3 μVP plus illumination with a light intensity of 5.5 mW/cm^2^, respectively. The lower parts in each panel indicate the expanded traces recorded from the uppermost part. Note that channel opening causes an upward defection. **(B)** Summary bar graph showing the effects of VP and VP plus illumination on the probability of BK_Ca_ channels that would be open (mean ± SEM; *n* = 11–13 for each bar). *Significantly different from control (*P* < 0.05), ^†^significantly different from control (*P* < 0.01), and **significantly different from VP (3 μM) plus illumination group (*P* < 0.01).

The results from our whole-cell experiments reflected that *I*_K(Ca)_ described above could be K^+^ flux through the large-conductance subtype of K_Ca_ channels, because VP-induced increase in *I*_K(Ca)_ was effectively suppressed by iberiotoxin or paxilline, yet not by TRAM-39. Therefore, in attempts to ascertain how VP interacts pertinently with ion-channel activity to modify *I*_K(Ca)_, the effects of VP on BK_Ca_-channel activity was also investigated in cell-attached or inside-out configuration. In cell-attached current recordings, cells were bathed in normal Tyrode's solution containing 1.8 mM CaCl_2_, the activity of BK_Ca_ channels measured at +60 mV relative to the bath can be readily detected in these cells. In particular, when VP (3 μM) was applied to the bath medium, the probability of channel openness was drastically raised (Figures 5A,B). The open probability of BK_Ca_ channels maintained at +60 mV in the control was found to be 0.014 ± 0.004 (*n* = 13). One minute after addition of VP (3 μM) to the bath, the channel open probability was significantly increased to 0.024 ± 0.007 (*n* = 13, *P* < 0.05). The channel activity was reduced to 0.018 ± 0.005 (*n* = 12) after washout of the agent. However, the amplitude of single-channel currents remained unaltered in the presence of VP. The single-channel conductance of BK_Ca_ channels in control cells was 146 ± 8 pS (*n* = 12), a value that did not differ significantly from that (147 ± 13 pS, *n* = 12, *P* > 0.05) obtained in the presence of VP (3 μM). Moreover, in continued presence of 3 μM VP, further exposure to illumination with light intensity of 5.5 mW/cm^2^ increased channel open probability further to 0.047 ± 0.011 (*n* = 11, *P* < 0.01). Subsequent addition of 1 μM paxilline reduced channel activity significantly (Figure 5B), as evidenced by a significant reduction in the channel open probability to 0.017 ± 0.005 (*n* = 11, *P* < 0.05).

### Effect of VP, VP Plus GMQ, and VP PF573228 on BK_**Ca**_-Channel Activity in GH_3_ Cells

**Figure 6:**
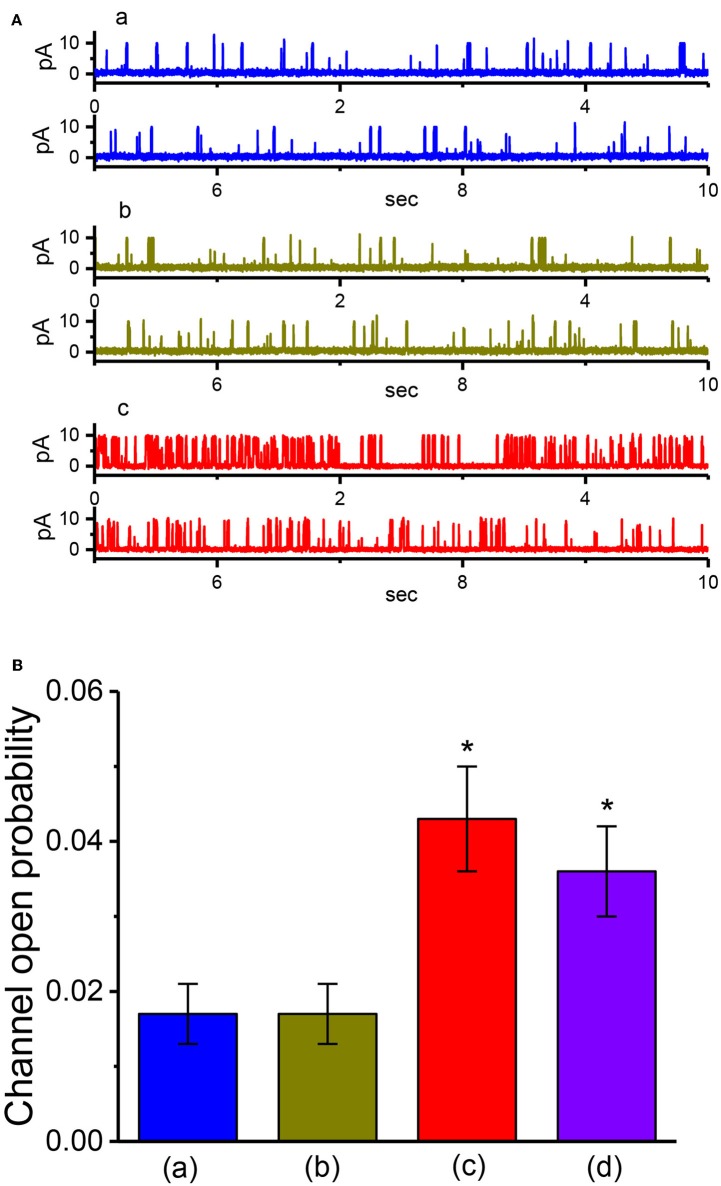
The activity of BK_Ca_ channels produced by VP, VP plus GMQ, or VP plus PF573228 under inside-out current recordings. In these experiments, cells were bathed in high-K^+^ solution which contained 1 μM Ca^2+^, the excised patch was maintained at +60 mV, and tested compound was applied to the bath. **(A)** BK_Ca_-channel current traces measured at +60 mV. a: control; b: 3 μM VP; c: 3 μM VP plus 3 μM GMQ. The open state of the channel is indicated by upward deflection. **(B)** Summary bar graph showing the effects of VP, VP plus GMQ, or VP plus PF573228 on the probability of BK_Ca_ channels that would be open (mean ± SEM; *n* = 12 for each bar). *Significantly different from control (i.e., in the absence of any agents; *P* < 0.05) and **significantly different from VP (3 μM) alone group (*P* < 0.05).

We also evaluated whether VP could exert any perturbation on BK_Ca_ channels in inside-out patch of the cell. Cells were immersed in high K^+^ solution containing 1 μM Ca^2+^, and the experiments were conducted in inside-out current recordings. As shown in Figure 6, when the excised patch was maintained at +60 mV, the addition of 3 μM VP to the intracellular leaflet of the inside-out patch did not modify the channel open probability. However, in continued presence of VP, further addition of either 2-guanidine-4-methylquinazoline (GMQ; 3 μM) or PF573228 (3 μM) was effective at raising the probability of BK_Ca_ channels. GMQ or PF573228 was previously reported to activate BK_Ca_ channels (So et al., 2011, 2018). The results thus showed the inability of VP to alter the probability of BK_Ca_-channel openings recorded from the excised patch of GH_3_ cells.

### Suppressive Effect of VP on L-Type Ca^2+^ Current (I_**Ca, L**_) in GH_3_Cells

**Figure 7:**
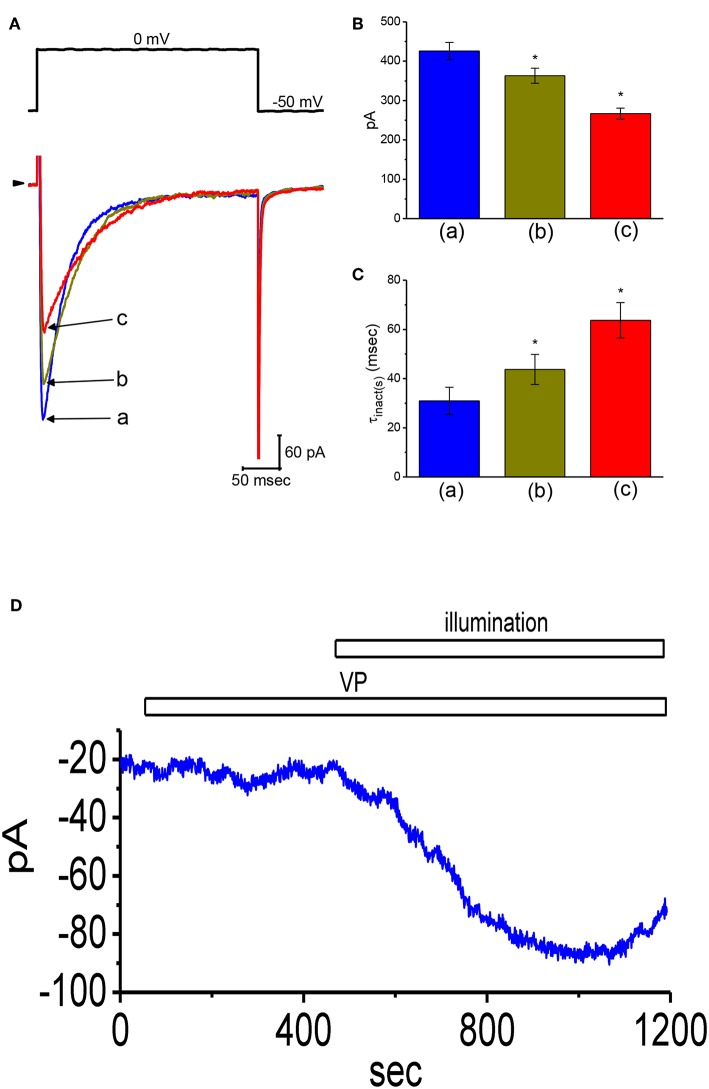
Effects of VP on L-type Ca^2+^ current (*I*_Ca,L_) and non-selective cation current (*I*_NS_) in GH_3_ cells. In this set of experiments, we bathed cells in normal Tyrode's solution containing 1.8 mM CaCl_2_, 1 μM TTX, and 10 mM TEA, and the pipette used was filled with Cs^+^-containing solution. **(A)** Original *I*_Ca,L_ traces elicited by membrane depolarization from −50 to 0 mV [indicated in the upper part of **(A)**]. a: control; b: 1 μM VP; c: 3 μM VP. Panels **(B)** and **(C)** depict summarized bar graphs showing the effect of VP on the peak amplitude of *I*_Ca,L_ and the slow component of inactivation time constant (τ_inact(s)_) for *I*_Ca,L_, respectively (mean ± SEM; *n* = 12 for each bar). **(D)** Change in the amplitude of *I*_NS_ obtained in the presence of VP and VP plus illumination. The examined cell was held at the level of −50 mV. The horizontal bar indicates addition of 3 μM VP or illumination with light intensity of 5.5 mW/cm^2^. *Significantly different from control (*P* < 0.01).

Whether VP exerts any perturbations on voltage-gated *I*_Ca,L_ in these cells was further studied, because any modifications in *I*_Ca,L_ magnitude can modify the level of intracellular Ca^2+^, thereby influencing either the activity of BK_Ca_ channels or *I*_K(Ca)_ amplitude (Wu et al., 1998a,b, 2003; Stojilkovic et al., 2010). As cells were exposed to different VP concentrations the peak amplitude of *I*_Ca,L_ elicited in response to single depolarizing step was progressively decreased (Figures 7A,B). The peak amplitude of *I*_Ca,L_ by membrane depolarization was suppressed by nifedipine (3 μM) and enhanced by Bay K 8644 (3 μM) (data not shown). Moreover, the addition of 3 μM VP suppressed the peak amplitude of *I*_Ca,L_ from 426 ± 22 to 267 ± 14 pA (*n* = 12, *P* < 0.05). Concomitant with this, the inactivation time constant of *I*_Ca,L_ became slowed (Figure 7C). The value for the slow component of *I*_Ca,L_ inactivation time constant (τ_inact(s)_) was prolonged to 63.7 ± 7.2 ms from a control value of 30.9 ± 5.6 ms (*n* = 12, *P* < 0.05). However, the fast component for the inactivation time constant of *I*_Ca,L_ did not differ significantly between the absence and presence of 3 μM VP. The results indicate that the presence of VP is capable of altering the amplitude and inactivation time course of *I*_Ca,L_ elicited in response to rapid membrane depolarization.

### Stimulatory Effect of VP on Non-selective Cation Current (I_**NS**_) in GH_3_Cells

In another set of experiments, we tested whether VP has any effects on *I*_NS_. The results showed that addition of VP (3 μM) did not have any effect on ion currents measured at the level of −50 mV. However, in continued presence of VP, as light illumination with intensity of 5.5 mW/cm^2^ was further applied, an inward current was progressively induced as the examined cell was voltage-clamp held at the level of −50 mV (Figure 7D). Similar findings were observed in eight different GH_3_ cells examined. As bathing solution was replaced with NMDG^+^ solution, this current could still be induced in the presence of VP plus light exposure. However, chlorotoxin (1 μM), an inhibitor of Cl^−^ channels, did not have any effects on photosensitized VP-induced inward currents. Therefore, consistent with previous observations reported from frog heart cells (Tarr et al., 1994), the inward currents seen in these cells exhibited to be the non-selective, yet not Cl^−^ ion-specific, nature of the ionic conductance.

### Inability of VP to Modify Voltage-Gated Na^+^ Current (I_**Na**_)

**Figure 8:**
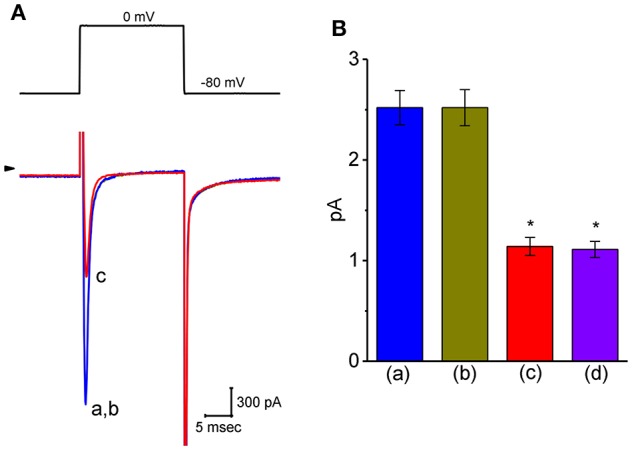
Effect of VP on voltage-gated Na^+^ current (*I*_Na_) in GH_3_ cells. The whole-cell current recordings made in this set of experiments were conducted in cells bathed in Ca^2+^-free Tyrode's solution containing 10 mM TEA and 0.5 mM CdCl_2_, and the recording pipette was filled with Cs^+^-containing solution. **(A)** Original *I*_Na_ traces elicited by rapid membrane depolarization. The voltage protocol used is indicated in the upper part and arrowhead refers to the zero-current level. a: control; b: 3 μM VP; c: 3 μM VP plus 3 μM A-803467. **(B)** Summary bar graph of the effect of VP, VP plus A-803467, and VP plus A-887826 on the peak amplitude of *I*_Na_ (mean ± SEM; *n* = 11 for each bar). The peak *I*_Na_ amplitude was measured at the beginning of short depolarizing pulse from a holding potential of −80 mV. *Significantly different from control (*P* < 0.05).

Another important type of voltage-gated ionic current (i.e., *I*_Na_) was also further examined to evaluate the possible modification of VP on this inward current. However, we were unable to detect any measurable change in the amplitude or gating of *I*_Na_ elicited by brief step depolarization (Figure 8). For example, the maximal peak amplitude of *I*_Na_ in the control was 2.52 ± 0.17 nA (*n* = 12), a value which did not differ significantly from that during cell exposure to 3 μM VP [2.52 ± 0.18 nA [*n* = 12, *P* > 0.05]]. In continued presence of 3 μM VP, subsequent addition of either A-803467 (3 μM) or A-887826 (3 μM) was potent in suppressing the peak *I*_Na_ in GH_3_ cells (Figure 8); however, no modification in *I*_Na_ inactivation in the presence of these two agents was demonstrated. A-803467 or A-887826 was previously reported to inhibit Na_V_1.8-encoded currents potently (Rush and Cummins, 2007; Zhang et al., 2010).

### Effect of VP on Bk_**ca**_-Channel Activity in Human 13-06-MG Glioma Cells

**Figure 9:**
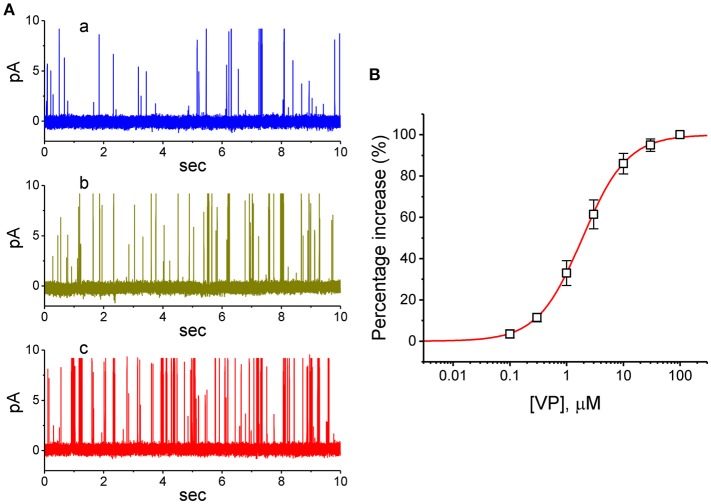
Stimulatory effect of VP on BK_Ca_-channel activity recorded from human 13-06-MG glioma cells. In this series of experiments, we bathed cells in normal Tyrode's solution and filled the recording pipette by using high K^+^-containing solution. **(A)** Representative BK_Ca_-channel currents taken under a holding potential of +60 mV relative to the bath. a: control; b: 1 μM VP; c: 3 μM VP. **(B)** Concentration-dependent relationship of VP effect on the probability of BK_Ca_-channel openings in 13-06-MG cells (mean ± SEM; *n* = 11 for each point). The examined cell was held at +60 mV relative to the bath, and the channel open probability during the exposure to 100 μM VP was taken to be 100%. The smooth line represents the least-squares fit of the data points to the sigmoidal Hill equation as described in **Materials and Methods**. The values of EC_50_, Hill coefficient, and maximal percentage increase of channel activity were estimated to be 1.9 μM, 1.1, and 100%, respectively.

VP has been recently reported to inhibit the proliferation of glioma cells (Al-Moujahed et al., 2017; Eales et al., 2018; Pellosi et al., 2019). In a final series of experiments, we therefore intended to investigate whether the presence of VP is also able to modulate ionic currents in another types of neoplastic cells (e.g., malignant glioma cells). As depicted in Figures 9A,B, in cell-attached measurements, the activity of BK_Ca_ channels in 13-06-MG glioma cells was robustly detected as described previously (Huang et al., 2015; Liu et al., 2015). Moreover, addition of VP to the bath increased the probability of BK_Ca_-channel openings in these cells. For example, as the cells were maintained at +60 mV relative to the bath, the exposure to 3 μM VP significantly elevated the channel open probability from 0.018 ± 0.007 to 0.038 ± 0.011 (*n* = 12, *P* < 0.05). Therefore, similar to that of GH_3_ cells described above, BK_Ca_-channel activity existing in 13-06-MG cells was indeed found to be sensitive to stimulation by VP. Apart from the inhibition of YAP-TEAD complex, VP-induced inhibition of cell proliferation in glioma (Eskelin et al., 2008; Eales et al., 2018) could, to some extent, be linked to its stimulatory action on BK_Ca_-channel activity.

## Discussion

The present investigation discloses important findings that VP produced a stimulatory effect on Ca^2+^-activated K^+^ current (*I*_K(Ca)_) in pituitary GH_3_ cells; however, it mildly depressed *I*_K(DR)_ amplitude with no modification on *I*_Na_ or *I*_K(M)_. The stimulatory effect on *I*_K(Ca)_ caused by VP was presumably related to changes in the level of intracellular Ca^2+^ concentrations. The results showing that removal of extracellular Ca^2+^ suppressed VP-induced increase in *I*_K(Ca)_ also suggest that extracellular Ca^2+^ and internal Ca^2+^ release both contribute to the increase in the amplitude of *I*_K(Ca)_ in these cells.

The EC_50_ value for either VP-induced stimulation of *I*_K(Ca)_ observed either in pituitary GH_3_ cells or increase in BK_Ca_-channel activity in 13-06-MG glioma cells was estimated to be 2.4 or 1.9 μM, respectively. These values are quite close to those that are either therapeutically achievable or required for the inhibitory actions on YAP-TEAD complex in different types of neoplastic cells (Faustino et al., 1997; Zhou et al., 2006; Feng et al., 2016; Gibault et al., 2016, 2017; Chen and Hu, 2017). As VP stimulated the *I*_K(Ca)_ amplitude within a few minutes in GH_3_ cells, it is therefore reasonable to elaborate that there is a conceivable link between the anti-neoplastic effects of this compound and its stimulatory actions on *I*_K(Ca)_, before it passes through the membrane (Marks et al., 2000).

By using rose bengal, a natural polyphenolic compound known to be another type of photosensitizer, previous studies have demonstrated that this compound challenged with or without photosensitization was able to modify membrane ionic current in GH_3_ cells along with mild damage on cell growth, although these cells were described to be relatively more resistant to photosensitized modification than other type of cells (Tarr et al., 1994; Valenzeno and Tarr, 1998, 2001). Similarly, findings from this study extended the observations and showed that VP was capable of stimulating *I*_K(Ca)_ in a concentration-dependent manner in GH_3_ cells. The illumination of VP could induce a non-selective cation current (Tarr et al., 1994), which appeared to be associated with the production of reactive oxygen species, and, in turn, increase the amplitude of *I*_K(Ca)_ further in GH_3_ cells (Valenzeno and Tarr, 1998; DeRosa and Crutchley, 2002). To what extent VP with or without illumination either modifies membrane potential or senses the photochemical modification remains to be further studied. It is also interesting to examine whether there are any other molecular target(s) which can regulate the VP-induced currents.

An earlier study has notably reported the capability of VP to dissipate mitochondrial inner transmembrane potential, hence decreasing the production of intracellular ATP (Belzacq et al., 2001). The pipette solution used in our whole-cell experiments contained 3 mM ATP; in particular, the amount of intracellular ATP is sufficient to fully suppress the activity of ATP-dependent K^+^ (K_ATP_) channels (Wu et al., 2000). Furthermore, VP-induced increase of outward currents observed in GH_3_ cells was unable to be reversed by subsequent application of either tolbutamide, a blocker of K_ATP_ channels, or TRAM-39, an inhibitor of IK_Ca_ channels (Huang et al., 2015; Chen P. C. et al., 2018; Chen T. S. et al., 2018; Liu et al., 2019a). As such, VP-induced increase in K^+^ outward currents conceivably is not associated with the activation of K_ATP_ or IK_Ca_ channels. Alternatively, in continued presence of VP, the ability of subsequent addition of dithiothreitol, a sulfhydryl reducing agent, to reverse VP-stimulated *I*_K(Ca)_ seems to lend credence to suggest a possible link to VP-induced production of free oxygen species (DeRosa and Crutchley, 2002; Morishita et al., 2016; Baskaran et al., 2018; Eales et al., 2018; Kim et al., 2018).

In our cell-attached current recordings, addition of VP into the bath increased the activity of BK_Ca_ channels significantly with no clear change in the single-channel conductance. These results can be stated as meaning that VP-stimulated channel activity results largely from the availability of intracellular Ca^2+^, despite inability of the VP molecules to act within the BK_Ca_-channel's central pore. However, VP-mediated activation of BK_Ca_ channels found in GH_3_ cells actually did not depend on increased availability of intracellular Ca^2+^owing to elevated Ca^2+^ flux through voltage-gated Ca^2+^ channels, given the present results showing that VP effectively suppressed the amplitude of *I*_Ca,L_ in GH_3_ cells. Additionally, in inside-out current recordings, as VP was applied to the intracellular surface of the excised patch, little or no change in the activity of BK_Ca_ channels was demonstrated; however, subsequent addition of GMQ or PF573228, known to stimulate BK_Ca_ channels, was effective at raising the channel open probability (So et al., 2011, 2018). Notably, it has been also demonstrated that BK_Ca_ channels are functionally expressed in either retinal pigment epithelial cells, vascular endothelial cells, or amniotic fluid-derived stem cells (Li et al., 2000; Schmidt-Erfurth and Hasan, 2000; Sheu et al., 2005; Hua et al., 2014; Hu et al., 2015; Liu et al., 2015, 2018, 2019a,b). Taking this into account, to what extent VP-induced changes in BK_Ca_-channel activity existing in these cells are intimately connected with its actions on sensory retina or pituitary adenoma during photodynamic therapy (Marks et al., 2000; Rahimipour et al., 2003; Rosenblatt et al., 2005; Hu et al., 2015; Nemes et al., 2016; Liu et al., 2018; Min et al., 2018; Iacono et al., 2019) remains to be imperatively investigated.

As being administrated, the VP molecules need to enter into cytosol or even nucleus, before being destined for having an interaction with YAP-TEAD complex (Brodowska et al., 2014; Feng et al., 2016; Gibault et al., 2016, 2017; Abe et al., 2018; Qin et al., 2019); hence, they must pass through surface membrane before entering the cell interior. From findings in this study, it is therefore tempting to speculate that the perturbations on membrane ion currents caused by VP would be required to precede its subsequent actions through suppression of YAP-TEAD complex. Moreover, another important determinants needed to be considered are the VP dose and the intensity of illumination, since the stimulatory effect of VP on *I*_K(Ca)_ could be highly related to generation of reactive oxygen species (Morishita et al., 2016; Baskaran et al., 2018; Eales et al., 2018; Kim et al., 2018). The sensitization sites for the modification caused by the VP molecules are thought to be probably at an intramembranous location near the inner surface. Upon cell exposure to VP particularly along with long-term light exposure, the reactive oxygen species would be excessively produced, and resultant byproducts of oxidative metabolism can then interact with membrane phospholipids and proteins that constitute ion transport pathways such as BK_Ca_ channels. Lastly, It will be worthy of being investigated regarding to what extent VP-mediated modifications on membrane ion channels reported herein is closely linked to the inhibition of VAP-TEAD complex followed by changes in proliferation or differentiation of different types of neoplastic or stem cells, given that the fact that such photosensitizers as sensitized photochemical modification in an array of tumors, including pituitary tumors and gliomas, have been widely utilized (Marks et al., 2000; Brown et al., 2004; Solban et al., 2006; Triesscheijn et al., 2006; Cole et al., 2008; Brodowska et al., 2014; Valero et al., 2015; Morishita et al., 2016; Nemes et al., 2016; Chen and Hu, 2017; Baskaran et al., 2018; Liao et al., 2018; Mulder et al., 2018; Chen et al., 2019; Li et al., 2019; Pellosi et al., 2019; Qin et al., 2019; Zhang et al., 2019). Alternatively, the efficacy of VP in inhibiting YAP/TEAD interaction was currently noted to remain debated (Lui et al., 2019). Regardless of the detailed mechanism of VP actions, it is pertinent to point out that VP-induced modifications on membrane ion channels shown herein should not be ignored and is conceivably responsible for effecting its therapeutic or adverse actions (Marks et al., 2000; Abe et al., 2018; Min et al., 2018; Iacono et al., 2019 Isildak et al., 2019).

## Data Availability

The raw data supporting the conclusions of this manuscript will be made available by the authors, without undue reservation, to any qualified researcher.

## Author Contributions

All authors listed have made a substantial, direct and intellectual contribution to the work, and approved it for publication.

### Conflict of Interest Statement

The authors declare that the research was conducted in the absence of any commercial or financial relationships that could be construed as a potential conflict of interest.

## Abbreviations

BK_Ca_ channel: large-conductance Ca^2+^-activated K^+^ channel
EC_50_: the concentration required for half-maximal stimulation
GMQ: 2-guanidine-4-methylquinazoline
*I-V*: current vs. voltage
*I*_Ca,L_: L-type Ca^2+^ current
IK_Ca_ channel: intermediate-conductance Ca^2+^-activated K^+^ channel
*I*_K(Ca)_: Ca^2+^-activated K^+^ current
*I*_K(DR)_: delayed-rectifier K^+^ current
*I*_K(M)_: M-type K^+^ current
*I*_Na_: voltage-gated Na^+^ current
*I*_NS_: non-selective cation current
TEA: tetraethylammonium chloride
TEAD: TEA domain transcription factor
τ_inact(s)_: slow component of inactivation time constant for ionic current
TTX: tetrodotoxin
VP: verteporfin (Visudyne®)
YAP: yes-associated protein.

## References

[B1] Abe T. Amaike Y. Shizu R. Takahashi M. Kano M. Hosaka T.. (2018). Role of YAP activation in nuclear receptor CAR-mediated proliferation of mouse hepatocytes. Toxicol. Sci. 165, 408–419. 10.1093/toxsci/kfy149 29893953

[B2] AlAmri M. Kadri H. Alderwick L. J. Jeeves M. Mehellou Y. (2018). The photosensitizing clinical agent verteporfin is an inhibitor of SPAK and OSR1 kinases. Chembiochem 4, 2072–2080. 10.1002/cbic.201800272 29999233

[B3] Al-Moujahed A. Brodowska K. Stryjewski T. P. Efstathiou N. E. Vasilikos I. Cichy J.. (2017). Verteporfin inhibits growth of human glioma *in vitro* without light activation. Sci. Rep. 7:7602. 10.1038/s41598-017-07632-8 28790340 PMC5548915

[B4] Baskaran R. Lee J. Yang S. G. (2018). Clinical development of photodynamic agents and therapeutic applications. Biomater. Res. 22:25. 10.1186/s40824-018-0140-z 30275968 PMC6158913

[B5] Belzacq A. S. Jacotot E. Vieira H. L. Mistro D. Granville D. J. Xie Z.. (2001). Apoptosis induction by the photosensitizer verteporfin: identification of mitochondrial adenine nucleotide translocator as a critical target. Cancer Res. 61, 1260–1264. 11245415

[B6] Brodowska K. Al-Moujahad A. Marmalidou A. Meyer Z. U. Horste M. Cichy J.. (2014). The clinically used photosensitizer verteporfin (VP) inhibits YAP-TEAD and human retinoblastoma cell growth *in vitro* without light activation. Exp. Eye Res. 124, 67–73. 10.1016/j.exer.2014.04.011 24837142 PMC4135181

[B7] Brown S. B. Brown E. A. Walker I. (2004). The present and further role of photodynamic therapy in cancer treatment. Lancet Oncol. 5, 497–508. 10.1016/s1470-2045(04)01529-3 15288239

[B8] Chen P. C. Ruan J. S. Wu S. N. (2018). Evidence of decreased activity in intermediate-conductance calcium-activated potassium channels during retinoic acid-induced differentiation in motor neuron-like NSC-34 cells. Cell. Physiol. Biochem. 48, 2374–2388. 10.1159/000492653 30114691

[B9] Chen T. S. Lai M. C. Hung T. Y. Lin K. M. Huang C. W. Wu S. N. (2018). Pioglitazone, a PPAR-γ activator, stimulates BK_Ca_ but suppresses IK_M_ in hippocampal neurons. Front. Pharmacol. 9:977 10.3389/fphar.2018.00977 30210346 PMC6123368

[B10] Chen Y. Hu Y. (2017). Photodynamic therapy for an iris metastasis from pulmonary adenocarcinoma. Photodiagn. Photodyn. Ther. 20, 246–247. 10.1016/j.pdpdt.2017.10.011 29107823

[B11] Chen Y. A. Lu C. Y. Cheng T. Y. Pan S. H. Chen H. F. Chang N. S. (2019). WW domain-containing proteins YAP and TAZ in the Hippo pathway as key regulators in stemness maintenance, tissue homeostasis, and tumorigenesis. Front. Oncol. 9:60. 10.3389/fonc.2019.00060 30805310 PMC6378284

[B12] Cole C. D. Liu J. K. Sheng X. Chin S. S. Schmidt M. H. Weiss M. H.. (2008). Hypericin-mediated photodynamic therapy of pituitary tumors: preclinical study in a GH_4_C_1_ rat tumor model. J. Neurooncol. 87, 255–261. 10.1007/s11060-007-9514-0 18228116

[B13] Deng W. Chen W. Clement S. Guller A. Zhao Z. Engel A.. (2018). Controlled gene and drug release from a liposomal delivery platform triggered by X-ray radiation. Nat. Commun. 9:2713. 10.1038/s41467-018-05118-3 30006596 PMC6045614

[B14] DeRosa M. C. Crutchley R. J. (2002). Photosensitized singlet oxygen and its applications. Coord. Chem. Rev. 233-234, 351–371. 10.1016/S0010-8545(02)00034-6

[B15] Eales K. L. Wilkinson E. A. Cruickshank G. Tucker J. H. R. Tennant D. A. (2018). Verteporfin selectively kills hypoxic glioma cells through iron-binding and increased production of reactive oxygen species. Sci. Rep. 8:14358. 10.1038/s41598-018-32727-1 30254296 PMC6156578

[B16] Eskelin S. Tommila P. Palosaari T. Kivelä T. (2008). Photodynamic therapy with verteporfin to induce regression of aggressive retinal astrocytomas. Acta Ophthalmol. 86, 794–799. 10.1111/j.1755-3768.2007.01151.x 18759802

[B17] Faustino M. A. Neves M. G. Vicente M. G. Cavaleiro J. A. Neumann M. Brauer H. D.. (1997). *Meso*-tetraphenylpoorphyrin dimer derivative as a potential photosensitizer in photodynamic therapy. Photochem. Photobiol. 66, 405–412. 9337611 10.1111/j.1751-1097.1997.tb03165.x

[B18] Feng J. Gou J. Jia J. Yi T. Cui T. Li Z. (2016). Verteporfin, a suppressor of YAP-TEAD complex, presents promising antitumor properties on ovarian cancer. Onco. Targets Ther. 9, 5371–5381. 10.2147/OTT.S109979 27621651 PMC5010158

[B19] Ghazai B. Machacek M. Shalaby M. A. Novakova V. Zimcik P. Makhseed S. (2017). Phthalocyanines and tetrapyrazinoporphyrazines with two cationic donuts: high photodynamic activity as a result of rigid spatial arrangement of peripheral substituents. J. Med. Chem. 60, 6060–6076. 10.1021/acs.jmedchem.7b00272 28558213

[B20] Gibault F. Bailly F. Corvaisier M. Coevoet M. Huet G. Melnyk P. (2017). Molecular features of the YAP inhibitor verteporfin: synthesis of hexasubstituted dipyrrins as potential inhibitors of YAP/TAZ, the downstream effects of the Hippo Pathway. ChemMedChem 12, 954–961. 10.1002/cmdc.201700063 28334506

[B21] Gibault F. Corvaisier M. Bailly F. Huet G. Melnyk P. Cotelle P. (2016). Non-photoinduced biological properties of verteporfin. Curr. Med. Chem. 23, 1171–1184. 10.2174/0929867323666160316125048 26980565

[B22] Hu Y. Chen Y. Chen L. (2015). Half-dosage and bolus injection photodynamic therapy for symptomatic circumscribed choroidal hemangioma: a case report. Photodiagn. Photodyn. Ther. 12, 526–529. 10.1016/j.pdpdt.2015.05.005 26007239

[B23] Hua R. Liu L. Li C. Chen L. (2014). Evaluation of the effects of photodynamic therapy on chronic central serous chorioretinopathy based on the mean choroidal thickness and the lumen area of abnormal choroidal vessels. Photodiagn. Phododyn. Ther. 11, 519–525. 10.1016/j.pdpdt.2014.07.005 25102163

[B24] Huang M. H. Huang Y. M. Wu S. N. (2015). The inhibition by oxaliplatin, a platinum-based anti-neoplastic agent, of the activity of intermediate-conductance Ca^2+^-activated K^+^ channels in human glioma cells. Cell. Physiol. Biochem. 37, 1390–1406. 10.1159/000430404 26488725

[B25] Iacono P. Toto L. Eliana C. Varano M. Parravano M. C. (2019). Pharmacotherapy of central serous chorioretinopathy: review of the current treatments. Curr. Pharm. Des. 24, 4864–4873. 10.2174/1381612825666190123165914 30674250

[B26] Isildak H. Schwartz S. G. Flynn H. W. (2019). Pharmacotherapy of myopic choroidal neovascularization. Curr. Pharm. Des. 24, 4853–4859. 10.2174/1381612825666190124102641 30674251

[B27] Kandoussi I. Lakhlili W. Taoufik J. Ibrahimi A. (2017). Docking analysis of verteporfin with YAP WW domain. Bioinformation 13, 237–240. 10.6026/97320630013237 28943729 PMC5602291

[B28] Karacorlu M. Karacorlu S. Ozdemir H. (2004). Nonarteritic anterior ischemic optic neuropathy after photodynamic therapy for choroidal neovascularization. Jpn. J. Ophthalmol. 48, 424–426. 10.1007/s10384-004-0078-7 15295679

[B29] Kim T. W. Moon J. W. Yu H. G. (2018). N-acetylcysteine protects against chorioretinal damage induced by photodynamic therapy for experimental choroidal neovascularization in a rat model. Phtodiagn. Photodyn. Ther. 23, 12–17. 10.1016/j.pdpdt.2018.04.006 29679669

[B30] Konstantinou E. K. Notomi S. Kosmidou C. Brodowska K. Al-Moujahed A. Nicolaou F.. (2017). Verteporfin-induced formation of protein cross-linked oligomers and high molecular weight complexes is mediated by light and leads to cell toxicity. Sci. Rep. 7:46581. 10.1038/srep46581 28429726 PMC5399488

[B31] Li H. F. Chen S. A. Wu S. N. (2000). Evidence for the stimulatory effect of resveratrol on Ca^2+^-activated K^+^ current in vascular endothelial cells. Cardiovasc. Res. 45, 1035–1045. 10.1016/S0008-6363(99)00397-1 10728430

[B32] Li Y. Wang S. Wei X. Zhang S. Song Z. Chen X.. (2019). Role of inhibitor of yes-associated protein 1 in triple-negative breast cancer with taxol-based chemoresistance. Cancer Sci. 110, 561–567. 10.1111/cas.13888 30467925 PMC6361558

[B33] Liao T. Wei W. J. Wen D. Hu J. Q. Wang Y. Ma B.. (2018). Verteporfin inhibits papillary thyroid cancer cells proliferation and cell cycle through ERK1/2 signaling pathway. J. Cancer. 9, 1329–1336. 10.7150/jca.21915 29721041 PMC5929076

[B34] Lin J. Gu Y. Du R. Deng M. Lu Y. Ding Y. (2014). Detection of EGFR mutation in supernatant, cell pellets of pleural effusion and tumor tissues from non-small cell lung cancer patients by high resolution melting analysis and sequencing. Int. J. Clin. Exp. Pathol. 7, 8813–8822. 25674250 PMC4313957

[B35] Lin M. W. Yang S. R. Huang M. H. Wu S. N. (2004). Stimulatory actions of caffeic acid phenethyl ester, a known inhibitor of NF-κB activation, on Ca^2+^-activated K^+^ current in pituitary GH_3_ cells. J. Biol. Chem. 279, 26885–26892. 10.1074/jbc.M400356200 15039450

[B36] Liu C. C. Huang Y. M. Wu S. N. Sze C. I. (2015). Correlation of mesenchymal differentiation and ion-channel activity in radiation-induced resistant human glioblastoma cell line. FASEB J. 29:1 Available online at: https://www.fasebj.org/doi/abs/10.1096/fasebj.29.1_supplement.844.1725561464

[B37] Liu L. Hu C. Chen L. Hu Y. (2018). Photodynamic therapy for symptomatic circumscribed choroidal hemangioma in 22 Chinese patients: a retrospective study. Photodiagn. Photodyn. Ther. 24, 372–376. 10.1016/j.pdpdt.2018.10.019 30381258

[B38] Liu Y. W. Fang Y. H. Su C. T. Hwang S. M. Liu P. Y. Wu S. N. (2019a). The biochemical and electrophysiological profiles of amniotic fluid-derived stem cells following Wnt signaling modulation cardiac differentiation. Cell Death Discov. 5:59. 10.1038/s41420-019-0143-0 30701091 PMC6349909

[B39] Liu Y. Y. Hsiao H. T. Wang J. C. Liu Y. C. Wu S. N. (2019b). Parecoxib, a selective blocker of cyclooxygenase-2, directly inhibits neuronal delayed-rectifier K^+^ current, M-type K^+^ current and Na^+^ current. Eur. J. Pharmacol. 844, 95–101. 10.1016/j.ejphar.2018.12.005 30529469

[B40] Lui J. W. Xiao S. Ogomori K. Hammarstedt J. E. Little E. C. Lang D. (2019). The efficacy of verteporfin as a therapeutic option in pre-clinical models of melanoma. J. Cancer. 10, 1–10. 10.7150/jca.27472 30662519 PMC6329844

[B41] Marks P. V. Belchetz P. E. Saxena A. Igbaseimokumo U. Thomson S. Nelson M. (2000). Effect of photodynamic therapy on recurrent pituitary adenomas: clinical phase I/II trial-an early report. Br. J. Neurosurg. 14, 317–325. 10.1080/026886900417298 11045196

[B42] Min J. Y. Lv Y. Mao L. Gong Y. Y. Gu Q. Wei F. (2018). A rodent model of anterior ischemic optic neuropathy (AION) based on laser photoactivation of verteporfin. BMC Ophthalmol. 18:304. 10.1186/s12886-018-0937-5 30466418 PMC6251118

[B43] Morishita T. Hayakawa F. Sugimoto K. Iwase M. Yamamoto H. Hirano D.. (2016). The photosensitizer verteporfin has light-independent anti-leukemic activity for Ph-positive acute lymphoblastic leukemia and synergistically works with dasatinib. Oncotarget 7, 56241–56252. 10.18632/oncotarget.11025 27494842 PMC5302911

[B44] Mulder C. L. Eijkenboom L. L. Beerendonk C. C. M. Braat D. D. M. Peek R. (2018). Enhancing the safety of ovarian cortex autotransplantation: cancer cells are purged completely from human ovarian tissue fragments by pharmacological inhibition of YAP/TAZ oncoproteins. Hum. Reprod. 34, 506–518. 10.1093/humrep/dey384 30597012

[B45] Nemes A. Fortmann T. Poeschke S. Greve B. Prevedello D. Santacroce A.. (2016). 5-ALA fluorescence in native pituitary adenoma cell lines: resection control and basis for photodynamic therapy (PDT)? PLoS ONE 11:e0161364. 10.1371/journal.pone.0161364 27583461 PMC5008746

[B46] Pellosi D. S. Paula L. B. de Melo M. T. Tedesco A. C. (2019). Targeted and synergic glioblastoma treatment: multifunctional nanoparticle delivering verteporfin as adjuvant therapy for temozolomide chemotherapy. Mol. Pharm. 16, 1009–1024. 10.1021/acs.molpharmaceut.8b01001 30698450

[B47] Qin X. Li J. Sun J. Liu L. Chen D. Liu Y. (2019). Low shear stress induces ERK nuclear localization and YAP activation to control the proliferation of breast cancer cells. Biochem. Biophys. Res. Commun. 510, 219–223. 10.1016/j.bbrc.2019.01.065 30685085

[B48] Rahimipour S. Ben-Aroya N. Ziv K. Chen A. Fridkin M. Koch Y. (2003). Receptor-mediated targeting of a photosensitizer by its conjugation to gonadotropin-releasing hormone analogues. J. Med. Chem. 46, 3965–3974. 10.1021/jm020535y 12954050

[B49] Renno R. Z. Terada Y. Haddadin M. J. Michaud N. A. Gragoudas E. S. Miller J. W. (2004). Selective photodynamic therapy by targeted verteporfin delivery to experimental choroidal neovascularization mediated by a homing peptide to vascular endothelial growth factor receptor-2. Arch. Ophthalmol. 122, 1002–1011. 10.1001/archopht.122.7.1002 15249365

[B50] Rosenblatt B. J. Shah G. K. Blinder K. (2005). Photodynamic therapy with verteporfin for pericapillary choroidal neovascularization. Retina 25, 33–37. 15655438 10.1097/00006982-200501000-00004

[B51] Rush A. M. Cummins T. R. (2007). Painful research: identification of a small-molecule inhibitor that selectively targets Na_V_1.8 sodium channels. Mol. Interv. 7, 192–195. 10.1124/mi.7.4.4 17827438

[B52] Sankaranarayanan S. Simasko S. M. (1996). Characterization of an M-like current modulated by thyrotropin-releasing hormone in normal rat lactotrophs. J. Neurosci. 16, 1668–1678. 8774435 10.1523/JNEUROSCI.16-05-01668.1996 PMC6578674

[B53] Schmidt-Erfurth U. Hasan T. (2000). Mechanisms of action of photodynamic therapy with verteporfin for the treatment of age-related macular degeneration. Surv. Ophthalmol. 45, 195–214. 10.1016/S0039-6257(00)00158-2 11094244

[B54] Sheu S. J. Wu S. N. Hu D. N. (2005). Stretch-stimulated activity of large conductance calcium-activated potassium channels in human retinal pigment epithelial cells. J. Ocul. Pharmacol. Ther. 21, 429–435. 10.1089/jop.2005.21.429 16386084

[B55] So E. C. Foo N. P. Ko S. Y. Wu S. N. (2019). Bisoprolol, known to be a selective β_1_-receptor antagonist, differentially but directly suppresses I_K(M)_ and I_K(erg)_ in pituitary cells and hippocampal neurons. Int. J. Mol. Sci. 20:657 10.3390/ijms20030657 PMC6386942 30717422

[B56] So E. C. Wang Y. Yang L. Q. So K. H. Lo Y. C. Wu S. N. (2018). Multiple regulatory actions of 2-guanidine-4-methylquinazoline (GMQ), an agonist of acid-sensing ion channel type-3, on ionic currents in pituitary GH_3_ cells and in olfactory sensory (Rolf B1.T) neurons. Biochem. Pharmacol. 151, 79–88. 10.1016/j.bcp.2018.02.027 29477572

[B57] So E. C. Wu K. C. Liang C. H. Chen J. Y. Wu S. N. (2011). Evidence for activation of BK_Ca_ channels by a known inhibitor of focal adhesion kinase, PF573228. Life Sci. 89, 691–701. 10.1016/j.lfs.2011.08.013 21925512

[B58] Solban N. Rizvi I. Hasan T. (2006). Targeted photodynamic therapy. Lasers Surg. Med. 38, 522–531. 10.1002/lsm.20345 16671102

[B59] Stojilkovic S. S. Tabak J. Bertram R. (2010). Ion channels and signaling in the pituitary gland. Endoc. Rev. 31, 845–915. 10.1210/er.2010-0005 20650859 PMC3365841

[B60] Tarr M. Arriaga E. Goertz K. K. Valenzeno D. P. (1994). Properties of cardiac I_leak_ induced by photosensitizer-generated reactive oxygen. Free Radic. Biol. Med. 16, 477–484. 7516303 10.1016/0891-5849(94)90125-2

[B61] Tekrony A. D. Kelly N. M. Fage B. A. Cramb D. T. (2011). Photobleaching kinetics of verteporfin and lemuteporfin in cells and optically trapped multilamellar vesicles using two-photon excitation. Photochem. Photobiol. 87, 853–861. 10.1111/j.1751-1097.2011.00933.x 21488879

[B62] Triesscheijn M. Baas P. Schellens J. H. Stewart F. A. (2006). Photodynamic therapy in oncology. Oncologist 11, 1034–1044. 10.1634/theoncologist.11-9-1034 17030646

[B63] Valenzeno D. P. Tarr M. (1998). GH_3_ cells, ionic currents and cell killing: photomodification sensitized by Rose Bengal. Photochem. Photobiol. 68, 519–526. 9796434

[B64] Valenzeno D. P. Tarr M. (2001). Calcium as a modulator of photosensitized killing of H9c2 cardiac cells. Phtochem. Photobiol. 74, 605–610. 10.1562/0031-8655(2001)074<0605:caamop>2.0.co;2 11683041

[B65] Valero V. III. Pawlik T. M. Anders R. A. (2015). Emerging role of Hpo signaling and YAP in hepatocellular carcinoma. J. Hepatocell Carcinoma 2, 69–78. 10.2147/JHC.S48505 27508196 PMC4918286

[B66] Wu S. N. Chern J. H. Shen S. Chen H. H. Hsu Y. T. Lee C. C.. (2017). Stimulatory actions of a novel thiourea derivative on large-conductance, calcium-activated potassium channels. J. Cell. Physiol. 232, 3409–3421. 10.1002/jcp.25788 28075010

[B67] Wu S. N. Chiang H. T. Shen A. Y. Lo Y. K. (2003). Differential effects of quercetin, a natural polyphenolic flavonoid on L-type calcium current in pituitary tumor (GH_3_) cells and neuronal NG108-15 cells. J. Cell. Physiol. 195, 298–308. 10.1002/jcp.10244 12652656

[B68] Wu S. N. Li H. F. Chiang H. T. (2000). Characterization of ATP-sensitive potassium channels functionally expressed in pituitary GH_3_ cells. J. Membr. Biol. 178, 205–214. 10.1007/s002320010028 11140276

[B69] Wu S. N. Li H. F. Jan C. R. (1998a). Regulation of Ca^2+^-activated nonselective cationic currents in rat pituitary GH_3_ cells: involvement in L-type Ca^2+^ current. Brain Res. 812, 133–141. 10.1016/s0006-8993(98)00964-0 9813284

[B70] Wu S. N. Li H. F. Jan C. R. Chen I. J. Lo Y. C. (1998b). Selective block by glyceryl nonivamide of inwardly rectifying K^+^ current in rat anterior pituitary GH_3_ cells. Life Sci. 63, PL281–PL288. 10.1016/s0024-3205(98)00447-0 9806231

[B71] Zhang Q. Guo Y. Yu H. Tang Y. Yuan Y. Jiang Y.. (2019). Receptor activity-modifying protein 1 regulates the phenotypic expression of BMSCs via the Hippo/Yap pathway. J. Cell. Physiol. 234, 13969–13976. 10.1002/jcp.28082 30618207 PMC13483059

[B72] Zhang X. F. Shieh C. C. Chapman M. L. Matulenko M. A. Hakeem A. H. Atkinson R. N. (2010). A-887826 is a structurally novel, potent and voltage-dependent Na_V_1.8 sodium channel blocker that attenuates neuropathic tactile allodynia in rats. Neuropharmacology 59, 201–207. 10.1016/j.neuropharm.2010.05.009 20566409

[B73] Zhou X. Chen B. Hoopes P. J. Hasan T. Pogue B. W. (2006). Tumor vascular area correlates with photosensitizer uptake: analysis of verteporfin microvascular delivery in the Dunning rat prostate tumor. Photochem. Photobiol. 82, 1348–1357. 10.1562/2006-03-25-ra-858 17421078

